# Mortality Associated With Acute Respiratory Infections Among Children at Home

**DOI:** 10.1093/infdis/jiy517

**Published:** 2018-08-28

**Authors:** Mauricio T Caballero, Alejandra M Bianchi, Alejandra Nuño, Adrian J P Ferretti, Leandro M Polack, Ines Remondino, Mario G Rodriguez, Liliana Orizzonte, Fernando Vallone, Eduardo Bergel, Fernando P Polack

**Affiliations:** 1 Fundacion INFANT, Argentina; 2 Secretaria de Salud de Lomas de Zamora, Buenos Aires, Argentina; 3 Secretaria de Salud de Florencio Varela, Buenos Aires, Argentina; 4 Region Sanitaria VI, Buenos Aires, Argentina; 5 Instituto de Efectividad Clinica y Sanitaria, Buenos Aires, Argentina

**Keywords:** Children, community mortality, acute respiratory infections, respiratory syncytial virus, influenza, verbal autopsy

## Abstract

**Background:**

Numerous deaths in children aged <5 years in the developing world occur at home. Acute respiratory infections (ARIs) are thought to play an important role in these deaths. Risk factors and pathogens linked to fatal episodes remain unclear.

**Methods:**

A case-control study among low-income children aged <5 years was performed in Buenos Aires, Argentina, to define risk factors and viral pathogens among those who died of ARI at home.

**Results:**

A total of 278 families of children aged <5 years (of whom 104 died and 174 were healthy controls) participated in the study. A total of 87.5% of ARI-associated deaths occurred among infants aged <12 months. The estimated mortality rate due to ARI among infants was 5.02 deaths/1000 live births. Dying at home from ARI was associated with living in a crowded home (odds ratio [OR], 3.73; 95% confidence interval [CI], 1.41–9.88), having an adolescent mother (OR, 4.89; 95% CI, 1.37–17.38), lacking running water in the home (OR, 4.39; 95% CI, 1.11–17.38), incomplete vaccinations for age (OR, 3.39; 95% CI, 1.20–9.62), admission to a neonatal intensive care unit (OR, 7.17; 95% CI, 2.21–23.27), and no emergency department visit during the ARI episode (OR, 72.32; 95% CI, 4.82–1085.6). The at-home death rate due to respiratory syncytial virus infection among infants was 0.26 deaths/100 live births and that due to influenza was 0.07 deaths/1000 live births.

**Conclusions:**

Social vulnerabilities underlie at-home mortality due to ARI. Mortality rates due to RSV and influenza virus infection are high among infants at home and are similar to those reported for hospitalized children.

## Teaser

Specific subgroups of children in socioeconomically vulnerable communities are at higher risk for dying at home from acute respiratory tract infection. This is the first study to provide at-home mortality rates due to respiratory syncytial virus and influenza A virus infection among infants.

The overwhelming majority of infant deaths occur in developing countries, and a significant number of these deaths occur at home [1, 2]. At-home deaths are often presumptively associated with acute respiratory infections (ARIs) and typically burden disenfranchised populations, in which families may not recognize life-threatening medical signs or may lack the means to act on them [3–5]. Preemptive actions are difficult to plan, since public health programs are hampered by the paucity of information on cause of death at home [6, 7]. The best available tools to date for this purpose, verbal autopsies (VAs), can be inconsistent in discriminating syndromic causes of infant mortality (such as ARI) and are unable by nature to identify microbial agents associated with fatal outcomes [8, 9]. For these reasons and many others, decreasing the burden of at-home deaths is challenging [10].

A number of pathogens have been identified as major drivers of pediatric mortality due to respiratory ailments [2, 11]. Yet, some of these fatal agents—respiratory viruses during ARI being a good example—infect previously healthy infants in industrialized countries without posing major threats to the infants’ survival [12]. These observations suggest that the causal factors of infant mortality at home exceed the etiologic agent(s) ultimately associated with demise. In fact, environmental, constitutional, and social factors may play a critical role in at-home deaths, specifically in those associated with ARI, affecting specific subgroups of families and infants in vulnerable communities [4, 13–15].

While the regions hosting vulnerable populations around the world are generally known, the characteristics identifying smaller groups or families in which a young child is at risk for dying at home from any cause and during ARI are unclear [6]. Therefore, a targeted allocation of public health or community resources to monitor and support those subgroups or families with young children at highest risk for death is difficult to accomplish. Moreover, the relative weights of social, biological, and medical care–related factors in fatal outcomes are also unclear. In addition, even in deaths presumptively associated with ARI, the role of viral pathogens such as respiratory syncytial virus (RSV) or influenza A virus, for which preventive interventions are under evaluation or available, remains unknown [6, 7, 10, 16].

Between 2014 and 2016, we conducted a case-control study in a population of infants and children aged <5 years and their families living in a low-income region in the outskirts of Buenos Aires, Argentina. The overwhelming majority of these children were infants <12 months of age. This study aimed to define risk factors for at-home mortality due to ARI in children aged <5 years. During 2016, we expanded our program to investigate the role of RSV, influenza virus, and other respiratory viruses in these fatal episodes. Our results suggest an important role for social vulnerability and provide, for the first time to our knowledge, information on at-home deaths associated with RSV, rhinovirus, human parainfluenza virus type 3, and influenza virus in a low-income region of the developing world.

## METHODS

### Study Population

This study is an age-matched, case-control, population-based study aimed to determine risk factors for community death due to ARI in a socioeconomically vulnerable district neighboring Buenos Aires. The case-control study was conducted between 1 April 2014 and 31 December 2016 in a catchment population of 50120 children aged <5 years without medical insurance. Thirty-five percent of these children lived in extreme poverty, in slums or very precarious settlements. Cases were defined as children dying at home without medical assistance and were detected using the Buenos Aires State Registration’s system, complemented by monthly review of death records at the local morgue and weekly monitoring of cemetery archives. Controls were the first living children matched by age (±3 months) who were identified by the research team in homes ≤300 m away while walking around the block. Depending on availability, a maximum of 2 controls were enrolled per case.

### Data Collection

Between 30 and 90 days after a child died, parents or direct relatives who lived at home with cases or controls were visited and interviewed by previously trained social workers, using a modified World Health Organization verbal autopsy (VA) instrument to determine cause of death [17]. ARI was assigned as the cause of death, using a mortality classification system derived from the *International Statistical Classification of Disease and Related Health Problems, Tenth Revision*, from the WHO [18]. This tool defines codes for diseases, disorders, injuries, and other health conditions and allows tracking of new diagnoses. For surviving controls, families were interviewed with the same instrument and, when discussing disease-related questions, were asked about the last episode of illness experienced by their living child. To ascertain risk factors for at-home mortality due to ARI, we obtained information on demographic, socioeconomic, and environmental factors and healthcare utilization, using a previously structured questionnaire. Instruments were in Spanish and pretested by state healthcare providers from the Argentina Ministry of Health. Data were stored in REDCap. The state’s institutional review board approved the study.

### Respiratory Viruses

To investigate viral etiologic agents of ARI in community deaths, we swabbed the nasopharynx of cases aged <5 years, who were routed to the local morgue between 1 May 2016 and 30 April 2017. While our case-control study covered 50120 children aged <5 years in the catchment population, deaths from 3 additional districts in the region were routed to the local morgue; therefore, the catchment population in the cause-of-death study was increased approximately 4.6 times. All swab specimens were collected ≤48 hours after death, frozen on site at −20°C, and stored at −80°C until processing.

RNA was extracted from nasopharyngeal samples, and real-time polymerase chain reaction (qPCR) analysis was used to assay for RSV, influenza A virus, influenza B virus, human parainfluenza virus type 3 (hPIV3), human metapneumovirus (hMPV), and human rhinoviruses (hRVs), as previously described [19].

### Statistical Analysis

Population numbers were derived from the national census in the catchment area to estimate community mortality rates due to ARI and specific viruses in children. Group characteristics for the case control study were compared using χ^2^ analysis and the Student *t* test where appropriate. Conditional logistic regression models were fitted for each explanatory variable separately to identify those associated with mortality. For each outcome, we fit a 3-level, multivariable, hierarchical, conditional logistic regression model, as previously described [19, 20]. Variables were grouped on the basis of their theoretical proximity to the death of the case. Variables with a *P* value of <.05 in the univariable analysis were considered in the multivariable model. The conditional logistic regression models were fitted in R 3.1.1, using the generalized linear models function.

## RESULTS

### Study Population

Our program follows a vulnerable population living in a large region in the southern outskirts of Buenos Aires. Within this vulnerable region, 35% of families live in extreme poverty in defined areas, comprising slums or precarious settlements.

**Figure 1.**
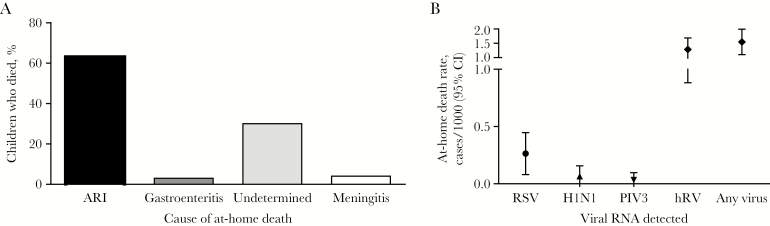
Distribution of the causes of at-home mortality determined by verbal autopsy (VA; *A*) and quantitative polymerase chain reaction (qPCR) of respiratory tract secretions (*B*). VA revealed that acute respiratory infections were the most frequent diagnosis, occurring in 63 children who died. qPCR analysis revealed that, among secretions testing positive for human rhinovirus (hRV), syncytial virus (RSV), influenza A(H1N1) virus, or parainfluenza virus type 3 (PIV3) RNA, at-home death rates were highest for hRV. CI, confidence interval.

No family we encountered had health insurance. The population in this region receives free health care from a network of primary care centers and can access 5 pediatric departments in general hospitals by public transportation. Despite the availability of medical care, at-home mortality rates due to ARI were high among infants, at 5.0 deaths/1000 live births (95% confidence interval [CI], 4.63–7.91) for the study period (Figure 1), with 92.8% of deaths occurring in postneonatal infants (99% of births in our region occur at hospitals). At-home mortality due to ARI among cases aged <5 years was estimated to be 1.13 deaths/1000 children (95% CI, 1.06–1.76). The duration of symptoms from ARI onset to death was 1–25 days (mean [±SD], 4.4 ± 4.9 days).

### Children Who Died Were Born in Challenging Home Environments in the Neighborhood

We first investigated whether at-home mortality due to ARI was influenced by factors that predated birth. Therefore, we compared the home environment of cases with ARI to that of surviving neighbors matched by age. Families of cases dying of ARI were more likely to live in precarious tin or mud houses, 6 times as likely to lack running water, and 2 times as likely to live in crowded conditions, compared with families of survivors (Table 1). Interestingly, when our explorations expanded to deaths of all causes, the same risk factors were apparent (Supplementary Table 1). But unlike in the group for whom ARI was diagnosed using VA, no receipt of state financial assistance through existing plans was identified as an additional risk factor for all-cause mortality.

**Table 1.** Univariable Analysis of Risk Factors for At-Home Death Due to Acute Respiratory Illness (ARI) Among Children Who Died of ARI (Cases) and Age-Matched Controls

Factor	Cases (n = 63)	Controls (n = 110)	OR (95% CI)	*P*
Socioeconomic characteristics				
Precarious household conditions				
Home made of tin or wood and with a dirt floor	38 (60.3)	45 (40.9)	**2.30 (1.16–4.58**)	**.018**
No running water	15 (23.8)	4 (3.6)	**6.14 (2.01–18.77**)	**.001**
Crowding (>3 person per bedroom)	39 (61.9)	33 (30)	**3.54 (1.74–7.19**)	**<.001**
Tobacco smoke inside	34 (53.9)	52 (47.3)	1.40 (.71–2.77)	.328
Vulnerable mother				
Adolescent age (<19 y)	11 (17.5)	6 (5.4)	**3.04 (1.11–8.30**)	**.030**
Incomplete primary education	52 (82.5)	83 (75.4)	2.11 (.91–4.87)	.080
Single marital status	10 (15.9)	16 (14.5)	1.15 (.45–2.94)	.763
Does not receive state aid	17 (26.9)	26 (23.6)	1.31 (.64–2.69)	.451
Previous interactions with healthcare system				
Incomplete vaccinations for age	16 (25.4)	9 (8.2)	**3.35 (1.42–7.92**)	**.006**
Never attended well-child visit	4 (6.3)	2 (1.8)	3.61 (.65–19.91)	.141
No or incomplete prenatal care	15 (23.8)	20 (18.2)	1.15 (.55–2.44)	.701
Biological and clinical characteristics				
Male sex	33 (52.4)	49 (44.9)	1.36 (.69–2.67)	.363
Prematurity	7 (11.1)	11 (10)	1.31 (.48–3.58)	.600
NICU admission	23 (36.5)	16 (14.5)	**4.84 (2.01–11.68**)	**<.001**
Low birth weight	6 (9.5)	4 (3.6)	3.08 (.73–12.99)	.125
Congenital malformation	6 (9.5)	4 (3.6)	3 (.85–10.63)	.089
Events during last disease episode				
Signs of moderate or severe illness	34 (53.9)	61 (55.4)	0.94 (.47–1.88)	.859
No PCC/ED visit	11 (17.5)	1 (0.9)	**18.21 (2.33–141.96**)	**.006**
Risk of SIDS	46 (73.02)	75 (68.2)	1.73 (.77–3.91)	.187

A total of 104 children died during the study, and 174 age-matched controls were enrolled from the surrounding neighborhood.

Bold values are statistically significant results, and gray values of the multivariable analysis mean the variable entry to the hierarchical regression (by step).

Abbreviations: CI, confidence interval; ED, emergency department; NICU, neonatal intensive care unit; OR, odds ratio; PCC, primary care clinic; SIDS, sudden infant death syndrome.

### Young Mothers of Cases Experienced Deficient Interactions With the Healthcare System Before Their Children Died

Next, we investigated whether maternal demographic information and maternal/child interactions with the healthcare system before the fatal illness affected the ultimate outcome (Table 1). Indeed, mothers of cases dying of ARI were more likely than mothers of surviving controls to be aged ≤18 years (Table 1). Rates of incomplete primary education (for 78%) and lack of or incomplete prenatal healthcare (for 20%) were worrisomely high in cases and controls but not significantly different between groups. In addition, one fourth of cases dying from ARI were not up to date in their immunizations, while this problem was present only in one twelfth of surviving controls (Table 1). Significant risk factors were similar when examining deaths of all causes (Supplementary Table 1).

### Biological Risk Factors for Fatal ARI

Vulnerable communities may have a greater proportion of children of frail constitution as compared to affluent neighborhoods. But whether children from these communities who die of ARI are constitutionally weaker and consequently more susceptible to ARI than surviving controls is unknown. If this hypothesis were correct, children at risk for ARI-associated mortality would be easily identifiable by history and physical examination. Therefore, we examined whether typical biological risk factors for ARI discriminated between groups (Table 1).

Comparisons revealed no differences in gestational age at birth, prematurity, sex, weight at birth, frequency of low birth weight, and having an asthmatic mother (Table 1). Interestingly, when the analysis was restricted to infants <12 months of age, breastfeeding was protective against at-home mortality (*P* < .011).

Despite the similar frequency of premature births in ARI cases and controls, neonatal intensive care unit (NICU) admissions were significantly more common in infant cases who died of ARI. This observation appears to be partly explained by the fact that the gestational age at birth of 61.5% of neonates admitted to NICUs was >37 weeks. NICU admissions of infants who later died in the community included a nonstatistically significant trend for more low birth weights, more congenital malformations (*P* = .09), and congenital infections (89% of which occurred in full-term infants, representing 25% of NICU admissions [*P* = .002] and including HIV infection in 8% of children who died).

### No Evidence for Risk Factors Associated With Sudden Infant Death Syndrome (SIDS) in Infant Cases Who Died of ARI at Home

A frequent cause attributed to at-home fatalities among infants in vulnerable regions is SIDS, which has often been hypothesized to be associated with viral ARI [21]. In fact, statistics in vulnerable regions often report rates of SIDS exceeding average international numbers by several fold [22]. These observations may favor resource allocation to targeted campaigns that, while always welcomed, compete for basic financial and human resources with other potentially preventive activities. Therefore, we investigated whether characteristic risk factors for SIDS were associated with ARI-associated mortality in our study. Interestingly, no differences were observed between cases and controls in the frequency of sleeping in the prone position (*P* = .214) and in sharing the bed with other household members (*P* = .08). The lack of a significant association persisted when combining both risk factors for at-home death due to ARI (Table 1) and all-cause mortality (Supplementary Table 1).

### Healthcare Use During Fatal ARI

We then asked control families whether they had consulted a healthcare professional during the last episode of any illness experienced by their child and compared their responses to those obtained from families of children with fatal ARI (Table 1). Not visiting a healthcare provider during the last episode of illness was significantly more frequent among children with ARI with fatal outcomes. Yet 82.5% of families with children who died of ARI reported that the child visited a physician during the fatal illness, suggesting that factors beyond receiving medical care influenced the infection outcome. Interestingly, parental perception of illness severity before the outcome in cases and controls did not differ between groups, with more than half of families perceiving that the ARI episode was moderate to severe (Table 1).

While VAs revealed no illness obvious to the parents of 36 of 104 cases (34.6%) and no immediate illness was recalled by families of 65 of 170 controls (38.2%), the use of healthcare facilities also differed between groups for all-cause mortality. The perception of disease severity remained similar in both groups (Supplementary Table 1).

### Hierarchical Multivariable Analysis of Risk Factors for Fatal ARI at Home

A hierarchical multivariable analysis of risk factors for at-home death due to ARI confirmed our previous observations. Exploratory analysis of socioeconomic variables revealed strong associations with a lack of running water, household crowding, having an adolescent mother, and having incomplete vaccinations for age (Table 2). Biological risk factors highlighted an independent role for previous NICU admissions, while the lack of healthcare use during the fatal case of ARI was strongly associated with a poor outcome (Table 2). Risk factors were similar for all-cause deaths, but a lack of state aid also negatively influenced disease evolution in this larger group (Supplementary Table 2).

**Table 2.** Hierarchical Multivariable Analyses of Risk Factors for Death Due to Acute Respiratory Illness

Factor	Level 1		Level 2		Level 3	
	OR (95% CI)	*P*	OR (95% CI)	*P*	OR (95% CI)	*P*
Precarious household conditions						
Home made of tin or wood and with a dirt floor	1.57 (.65–3.81)	.316	1.49 (.55–4.01)	.430	2.27 (.68–7.51)	.179
No running water	**4.39 (1.11–17.38**)	**.035**	**5.57 (1.13–27.47**)	**.035**	1.50 (.25–8.99)	.657
Crowding (>3 person per bedroom)	**3.73 (1.41–9.88**)	**.008**	**4.24 (1.42–12.67**)	**.010**	**6.69 (1.51–29.64**)	**.012**
Adolescent mother (age <19 y)	**4.89 (1.37–17.38**)	**.014**	3.50 (.84–14.57)	.085	5.33 (.82–34.52)	.079
Incomplete vaccination	**3.39 (1.20–9.62**)	**.021**	3.12 (.99–9.84)	.052	**6.84 (1.48–31.65**)	**.014**
NICU admission	…		**7.17 (2.21–23.27**)	**.001**	**13.48 (2.96–61.35**)	**.001**
No PCC/ED visit during last illness	…		…		**72.32 (4.82–1085.6**)	**.002**

Bold values are statistically significant results, and gray values of the multivariable analysis mean the variable entry to the hierarchical regression (by step).

Abbreviations: CI, confidence interval; ED, emergency department; NICU, neonatal intensive care unit; OR, odds ratio; PCC, primary care clinic.

### hRV and RSV in Children Aged <5 years Who Died of ARI at Home

Finally, given the high burden of ARI diagnosed by VA in at-home deaths, we investigated the role of respiratory viruses in deaths. For this purpose, we assayed respiratory secretions, using nasopharyngeal swab specimens obtained from cases aged <5 years and brought to the regional morgue within 12 hours of death and before burial, between May 2016 and April 2017 (see methods; Figure 1).

Curiously, hRVs were detected throughout the year (except in the summer); 45 swab specimens were positive for hRV and, in some instances, were associated with other viruses. However, we did not find differences in the rates of RV detection between cases who died of ARI and those who died of other causes (*P* = not significant). RSV was identified in swab specimens from 11 cases aged <5 years, including 8 aged <1 year (ie, infants) and 3 aged 1–5 years. Five of these cases were infected with RSV subgroup A, and 6 were infected with RSV subgroup B. In addition, influenza A(H1N1) virus was present in swab specimens from 2 infant cases, and hPIV3 was detected in the swab specimen from another infant case. Six cases had >1 pathogen detected in swab specimens, of whom 5 tested positive for hRV and RSV (n = 4) or hRV and influenza virus (n = 1) and 1 tested positive for RSV, influenza A(H1N1) virus, and hRV. RSV, influenza A(H1N1) virus, and hPIV3 were identified only during the respiratory season, between May and August. Neither hMPV nor influenza B virus RNA were detected in our cohort.

In summary, RSV was present in 11 of 114 cases (9.6%) aged <5 years over 1 year, in 11 of 37 samples (29.7%) obtained during the respiratory season, and in 11 of 53 samples (20.7%), collected over 1 year, in which we were able to detect RNA from at least 1 viral respiratory pathogen. While hRV is often detected in asymptomatic infants and children and, therefore, has a difficult-to-define role, RSV is routinely a pathogen/copathogen and is not expected to colonize the respiratory tract [23]. Therefore, among infants in our catchment population, the at-home mortality rate associated with the detection of RSV during ARI was estimated at 0.26 deaths/1000 live births (95% CI, .08–.45). Yet when we investigated the specific areas of the study region where infants died with RSV infection, we found that 78% of deaths occurred among infants in vulnerable slums and settlements, suggesting that fatalities cluster preferentially in the poorest districts (where the infant at-home mortality rate associated with RSV reached 1.4 deaths/1000 live births) [19]. In our study region, the at-home mortality rate due to influenza A(H1N1) virus among infants was lower, at 0.07 deaths/1000 live births (95% CI, .025–.16).

## DISCUSSION

It is widely assumed that infants in low-income regions of poor nations, as an indivisible category, are vulnerable and at risk for early death. This article represents an attempt to discriminate subgroups of children in at-risk communities who have a higher risk of dying of ARI. Our observations suggest that the specific home environment hosting the baby after birth and the family’s ability to leverage the public healthcare system and follow simple recommendations are critical determinants of the children’s fate. Dying infants with ARI may not be obviously sicker during their fatal illness or necessarily frailer than their neighbors before contracting disease. But they more often lack basic living conditions, and perhaps their families cannot master certain skills required for them to overcome disease [3, 5, 14, 15].

In our previous studies in these vulnerable communities, in-hospital deaths due to ARI did not preferentially affect a subgroup of infants in the slums and settlements. But here, at-home deaths did. In fact, deaths at home due to ARI appear to involve a different subgroup of children than pediatric deaths at hospitals in the same region. At hospitals, clinical complications (including pneumothoraxes and secondary bacterial infections) and comorbidities are the main secondary drivers of mortality [12, 19]. In the community, the list of social cofactors associated with death is vast. Worrisomely, and as observed in other studies of all-cause community mortality, medical care was available and provided to approximately 80% of children who died, but it was unable to prevent death [4, 5]. Evidently, some healthcare providers may have failed to comprehend the severity of the disease and risk factors and did not admit these children to the hospital preemptively. Unfortunately, our questionnaire failed to address whether parents received and/or understood alarm signs and whether they were instructed on how to proceed if they identified ≥1 of them. In fact, while parents clearly described to us signs of disease during the last days of their children’s lives, later in the interview they often reported that their children were perfectly healthy and suddenly died. These discrepancies and the absence of differences between cases and controls in the perceived severity of clinical signs during illness may reflect a lack of parental awareness of the gravity of the disease [5, 24].

An important observation involving children dying from ARI in this community is that a significant proportion, particularly during the respiratory illness season, had RNA from at least 1 viral pathogen detected in respiratory secretions. This is striking because mortality due to ARI among children living in middle-class areas of developing countries and in children from industrialized countries is very infrequent [25]. In fact, a study in 2009 estimated that the annual number of infant deaths due to RSV infection in the United States ranged from 56 to 121 in a large inpatient data set. More than 75% of these infants had comorbidities [12]. If the mortality rate from the community we studied was applied to the US, there would be an estimated 1040 at-home fatalities due to RSV infection annually. It is obvious that there is more to at-home deaths than viral infection. All this said, to our knowledge, our study is the first to provide primary data about the impact of RSV and influenza A(H1N1) virus infections on at-home deaths among infants. Rates of RSV-associated at-home mortality, examined for the first time by qPCR detection of the virus in nasopharyngeal secretions from dead children, shows that RSV kills children at home at a frequency similar to that in hospitals (0.26 deaths/1000 live births during 2016 and 0.3 deaths/1000 live births during 2011–2013, respectively). We also found that there is a population of children in this low-income region of Buenos Aires who are extremely vulnerable to these viruses, with a mortality rate exceeding previous indirect estimations of RSV-associated deaths [19].

Our study has limitations. First, VA is a useful instrument, particularly when administered in combination with autopsies, but VA has important inherent weaknesses when used alone [26, 27]. For instance, information about medical events leading to the child’s death are based heavily on parental recall of specific clinical signs and symptoms. In fact, particularly for syndromic causes of death, such as ARI, the sensitivity and specificity of VA are significantly lower than for events like snake bites or unintentional injuries [8, 28, 29].

Second, nasopharyngeal swabs cannot access the distal airways where viral pathogens cause disease. And preliminary data from hospital studies comparing upper and lower respiratory tract isolates in fatal cases suggest that these sites do not always match [30]. While this does not imply that pathogens isolated in the upper airway lack a role in respiratory illness, a more complex interpretation of the interplay between multiple pathogens in respiratory diseases may be necessary in coming years. For this purpose, in 2016 we extended our program to obtain lung sections and nasopharyngeal swab specimens from all fatal cases. In addition, endemic pathogens, genetic backgrounds, dietary and social habits, and environmental factors in different regions of the world may influence the translatability of our findings. Yet previous studies describing hospital deaths due to these viruses in Africa, Asia, and Latin America were remarkably similar, suggesting that some of this information may be translatable to other geographic regions [31, 32]. We enrolled >95% of families experiencing fatalities in children during the study period. Despite our strategies, some deaths avoid all registries bypassing state control. We were able to interview 2 of these families. These children had home deliveries, no demographic registry, no death certificate, and home burials.

In summary, serious social vulnerabilities underlie community mortality due to ARI in developing countries. Unfortunately, at present, in 2018, “the calamity of the rightless is not that they are deprived of life, liberty, and the pursuit of happiness, or of equality before the law and freedom of opinion—formulas which were designed to solve problems within given communities—but that they no longer belong to any community whatsoever” [33p295–6]. Precarious, crowded homes lacking running water house adolescent mothers who may not benefit from state support or leverage basic but critical public health interventions as often as their neighbors. These families are at risk for experiencing poor outcomes when their children are infected with respiratory pathogens. Home mortality rates for infants due to RSV and influenza virus are high and exceed, logarithmically, those reported for hospitalized children in industrialized countries,.

## Supplementary Data

Supplementary materials are available at *The Journal of Infectious Diseases* online. Consisting of data provided by the authors to benefit the reader, the posted materials are not copyedited and are the sole responsibility of the authors, so questions or comments should be addressed to the corresponding author.

Supplementary Table 1

Supplementary Table 2

Supplementary Form

## Notes

***Financial support.*** This work was supported by the Bill and Melinda Gates Foundation (grant OPP1157162 to F. P. P.).

***Potential conflicts of interest.*** F. P. P. was a consultant for Novavax, Bavarian Nordic, Regeneron, IQVIA RDS, and Merck and a member of the advisory board of Janssen, Pfizer, and Sanofi during the conduct of the study. All other authors report no potential conflicts. All authors have submitted the ICMJE Form for Disclosure of Potential Conflicts of Interest. Conflicts that the editors consider relevant to the content of the manuscript have been disclosed.
