# Supplementary material for: Mortality Associated With Acute Respiratory Infections Among Children at Home
Source: J Infect Dis. 2018 Aug 28;219(3):358–64. doi: 10.1093/infdis/jiy517 (PMC6325348; doi:10.1093/infdis/jiy517)
Supplement: Supplementary Table 1 [file jiy517_suppl_supplementary_table_1.pdf]

Supplementary table 1. Univariable analysis: Risk Factors for community death in under 5 years old children.

	Cases (n=104) n/N (%)	Controls (n=174) n/N (%)	Odds ratio (95% IC)	*p*
**Socioeconomic**				
Precarious home				
Home made of tin or wood and dirt floor	50/104 (48.1)	61/174 (35.1)	1.73 (0.99 - 3.02)	0.052
No running water	21/104 (20.2)	4/174 (2.3)	**6.41 (2.12 - 19.47)**	**0.001**
Crowding (more than 3 person per bedroom)	54/104 (51.9)	54/174 (31.03)	**2.17 (1.27 - 3.68)**	**0.004**
Tabacco smoke inside	49/104 (47.1)	64/174 (36.8)	1.73 (0.98 - 3.08)	0.058
Vulnerable mother				
Adolescent mother (<19 years)	21/104 (20.2)	7/174 (4.02)	**5.04 (2.13 - 11.95)**	**<0.001**
Incomplete primary maternal education	83/104 (79.8)	130/174 (74.7)	1.72 (0.90 - 3.31)	0.101
Single mother	21/104 (20.2)	23/174 (13.2)	1.60 (0.78 - 3.27)	0.195
Does not receive state aid	37/104 (35.4)	38/174 (21.8)	**2.40 (1.33 - 4.32)**	**0.003**
Previous interactions with the health care system				
Incomplete vaccinations for age	22/104 (21.1)	18/174 (10.3)	**2.47 (1.23 - 4.98)**	**0.011**
Never brought child for well-child visit	6/104 (5.8)	3/174 (1.7)	3.08 (0.73 - 12.99)	0.125
No or incomplete prenatal care	30/102 (29.4)	42/172 (24.4)	1.21 (0.67 - 2.20)	0.512
**Biological characteristics**				
Male sex	53/104 (50.9)	81/174 (46.5)	1.28 (0.77 - 2.15)	0.342
Prematurity	17/104 (16.4)	20/174 (11.49)	1.70 (0.82 - 3.50)	0.151
NICU admission	36/104 (34.6)	31/174 (17.8)	**3.16 (1.67 - 5.99)**	**<0.001**
Low birth weight (LBW)	16/104 (15.4)	11/174 (6.3)	**3.10 (1.14 - 8.45)**	**0.027**
Congenital malformation	6/104 (5.8)	4/174 (2.3)	3 (0.85 - 10.63)	0.089
**Events during last disease episode**				
Signs of moderate or severe illness	36/104 (34.6)	65/174 (37.4)	0.81 (041 - 1.58)	0.537
No PCC/ER visit during last illness	22/104 (21.1)	2/174 (1.1)	**14.20 (3.26 - 61.79)**	**<0.001**
Risk for sudden infant death syndrome	72/104 (69.2)	109/174 (62.6)	1.53 (0.85 - 2.78)	0.157
