# Supplementary material for: Mortality Associated With Acute Respiratory Infections Among Children at Home
Source: J Infect Dis. 2018 Aug 28;219(3):358–64. doi: 10.1093/infdis/jiy517 (PMC6325348; doi:10.1093/infdis/jiy517)
Supplement: Supplementary Table 2 [file jiy517_suppl_supplementary_table_2.pdf]

Supplementary table 2. Hierachical multivariable analysis: Risk Factors community death in under 5 years old children

	Level 1		Level 2		Level 3	
	**Odds ratio (95% IC)**	***p***	**Odds ratio (95% IC)**	***p***	**Odds ratio (95% IC)**	***p***
No running water	3.32 (0.89 - 12.42)	0.074	3.79 (0.90 - 15.96)	0.069	3 (0.61 - 14.56)	0.173
Crowding (more than 3 person per bedroom)	**3.61 (1.76 - 7.41)**	**<0.001**	**4.27 (1.89 - 9.69)**	**0.001**	**4.05 (1.69 - 9.69)**	**0.002**
Adolescent mother (<19 years)	**7.18 (2.46 - 20.95)**	**<0.001**	**7.08 (2.30 - 21.80)**	**0.001**	**8.37 (2.25 - 31.11)**	**0.002**
Does not receive state aid	**4.63 (2.10 - 10.21)**	**<0.001**	**6.35 (2.62 - 15.40)**	**<0.001**	**6.39 (2.50 - 16.36)**	**<0.001**
Incomplete vaccination	**2.49 (1.03 - 5.99)**	**0.042**	**2.68 (1.01 - 7.13)**	**0.048**	**3.91 (1.24 - 12.27)**	**0.019**
NICU admission			**5.22 (1.96 - 13.89)**	**0.001**	**6.64 (2.23 - 19.73)**	**0.001**
Low birth weight			1.57 (0.32 - 7.64)	0.576	1.59 (0.32 - 7.93)	0.572
No PCC/ER visit during last illness					**16.27 (2.74 - 96.57)**	**0.002**
