# Supplementary material for: Mortality Associated With Acute Respiratory Infections Among Children at Home
Source: J Infect Dis. 2018 Aug 28;219(3):358–64. doi: 10.1093/infdis/jiy517 (PMC6325348; doi:10.1093/infdis/jiy517)
Supplement: Supplementary Form [file jiy517_suppl_supplementary_form.pdf]

FICHA	MORTALIDAD INFANTIL DOMICILIARIA<br>Fundación Infant - Versión 2.1 - Octubre 2016	Florencio Varela ☐<br>Lomas de Zamora ☐

DATOS DEL ENTREVISTADO — A

DATOS PERSONALES — I

	¿Cuál fue la fecha del Fallecimiento de el/la niño?	☐☐ / ☐☐ / ☐☐☐☐
1.	Marcar con una cruz las personas que participan de la entrevista en relación al niño fallecido.<br>(Marque todas las que corresponda).	☐ Madre ☐ No familiar<br>☐ Padre ☐ Otro menor de 12 años<br>☐ Tío/a ☐ Otro mayor de 12 años<br>☐ Hermano/a ☐ Omite<br>☐ Abuelo/a ☐ Ns/Nc
2.	¿Quién es el/la principal interlocutor/a?	☐ Madre ☐ No familiar<br>☐ Padre ☐ Otro menor de 12 años<br>☐ Tío/a ☐ Otro mayor de 12 años<br>☐ Hermano/a ☐ Omite<br>☐ Abuelo/a ☐ Ns/Nc
3.	En caso en que la madre del niño fallecido no fue la principal interlocutora, explicar el principal motivo.	☐ Omite ☐ Ns/Nc
4.	¿Cuál es el mayor nivel educativo alcanzado de el/la principal interlocutor/a?	☐ Primaria incompleta<br>☐ Primaria completa<br>☐ Secundaria incompleta<br>☐ Secundaria completa<br>☐ Terciario/Universitario incompleto<br>☐ Terciario/Universitario completo<br>☐ Omite ☐ Ns/Nc
5.	El principal interlocutor, ¿se encontraba presente en el evento de muerte?	☐ Si ☐ No<br>☐ Omite ☐ Ns/Nc

ANTECEDENTES DEL NIÑO — B

ANTECEDENTES PERINATOLOGICOS — I

1.	¿Cuál fue la fecha del nacimiento de el/la niño/a?	☐☐ / ☐☐ / ☐☐☐☐
2.	¿Qué número de orden era el/la niño/a?	☐☐<br>☐ Omite ☐ Ns/Nc

Identificación del Paciente: __ __ - __ __ - __ __ - __ __ __ - __ __

Codificacion: dos letras nombre, dos letras apellido y fecha de nacimiento

FICHA	MORTALIDAD INFANTIL DOMICILIARIA<br>Fundación Infant - Versión 2.1 - Octubre 2016	Florencio Varela ☐<br>Lomas de Zamora ☐

**3.**	¿Tenía documento de identidad antes de fallecer?	☐ Si ☐ No<br>☐ Omite ☐ Ns/Nc
**4.**	¿Cuándo le eligieron el nombre?	☐ Antes de nacer ☐ Después de nacer<br>☐ Omite ☐ Ns/Nc
**5.**	¿Dónde nació?	☐ Hospital público ☐ Centro de salud<br>☐ Hospital privado ☐ Obra social<br>☐ En la casa ☐ Otro ↓<br>☐ Omite<br>☐ Ns/Nc
**6.**	En caso de haber nacido en una institución, escribir el nombre:	
**7.**	Lugar de nacimiento:	Ciudad:<br>País:<br>☐ Omite ☐ Ns/Nc
**8.**	¿Cuál era el sexo de el/la niño/a?	☐ Femenino ☐ Masculino
**9.**	El embarazo del niño/a ¿fue único o múltiple?	☐ Único ☐ Múltiple, número ☐<br>☐ Omite ☐ Ns/Nc
**10.**	¿Cómo fue el parto?	☐ Vaginal ☐ Cesárea, Motivo ↓<br>☐ Omite<br>☐ Ns/Nc
**11.**	¿Cuantas semanas de gestación tenía al nacer?	☐☐ semanas<br>☐ Omite ☐ Ns/Nc
**12.**	Si nació con menos de 38 semanas de gestación, ¿recibió la madre corticoides?	☐ Si ☐ No<br>☐ Omite ☐ Ns/Nc
**13.**	¿Cuánto pesó el/la niño/a al nacer?	☐☐☐☐ gramos<br>☐ Omite ☐ Ns/Nc
**14.**	¿Cuánto midió el/la niño/a al nacer?	☐☐ cm<br>☐ Omite ☐ Ns/Nc

Identificación del Paciente: __ __ - __ __ - __ __ - __ __ __ - __ __

Codificacion: dos letras nombre, dos letras apellido y fecha de nacimiento

**15.**	¿Cuál fue el perímetro cefálico de el/la niño/a al nacer?	☐☐ cm<br>☐ Omite ☐ Ns/Nc
**16.**	Anotar cuál fue al Apgar al minuto y a los cinco minutos de nacer	☐☐ 1 minuto ☐☐ 5 minutos<br>☐ Omite ☐ Ns/Nc
**17.**	¿Requirió el/la bebé internación en neonatología al nacer o antes del alta de la maternidad?	☐ Si ☐ No<br>☐ Omite ☐ Ns/Nc
	**(Si la respuesta es No, Omite o Ns/Nc pasar a la pregunta 23)**	
**18.**	Diagnóstico al ingreso en neonatología: marque todos los que corresponda.	☐ Sepsis ☐ Prematurez<br>☐ Problemas pulmonares ☐ Otro, especificar ↓<br>☐ Omite<br>☐ Ns/Nc
**19.**	¿Tuvo otras enfermedades o complicaciones durante la internación en neonatología?	☐ Si, cual/es? → ______<br>☐ No<br>☐ Omite ☐ Ns/Nc
**20.**	¿Requirió oxígeno en la internación (incluyendo ARM)?	☐ Si → ☐☐ días<br>☐ No<br>☐ Omite ☐ Ns/Nc
**21.**	¿Requirió asistencia respiratoria mecánica?	☐ Si → ☐☐ días<br>☐ No<br>☐ Omite ☐ Ns/Nc
**22.**	Al alta de neonatología, ¿requirió oxígeno suplementario?	☐ Si ☐ No<br>☐ Omite ☐ Ns/Nc
**23.**	¿Tuvo el/la niño/a cuando nació diagnóstico de infección perinatal confirmada?	☐ Si, cual/es? → ☐ HIV<br>☐ Sífilis<br>☐ Chagas<br>☐ Toxoplasmosis<br>☐ Citomegalovirus<br>☐ Estreptococo B<br>☐ Otro, especificar ↓<br>☐ No ______<br>☐ Omite ☐ Ns/Nc

**24.**	Luego del nacimiento y hasta el alta de la maternidad, ¿la internación fue conjunta madre- hijo/a?	☐ Si ☐ No<br>☐ Omite ☐ Ns/Nc
**25.**	Durante la internación conjunta ¿cómo se alimentó el/la niño/a?	☐ Pecho exclusivo<br>☐ Alimentación solo mamadera suplemento de leche (NO LECHE HUMANA)<br>☐ Pecho más complemento<br>☐ Omite ☐ Ns/Nc
**26.**	¿Fue amamantado el/la niño/a dentro del plazo de una hora de su nacimiento?	☐ Si ☐ No<br>☐ Omite ☐ Ns/Nc
**27.**	¿Cómo puede describir que era su niño/a durante los primeros días de vida (marque todo lo que corresponda)?	☐ Tranquilo ☐ Irritable<br>☐ Dormilón ☐ Siempre estaba con hambre<br>☐ Otro: Especificar ____<br>☐ Omite ☐ Ns/Nc
**28.**	¿Quién estuvo cuidándolo/a principalmente en la primera semana de vida?	☐ La mama ☐ La mama con el papa<br>☐ La mamá con ayuda permanente<br>☐ Otro: Especificar ____<br>☐ Omite ☐ Ns/Nc
**29.**	¿Donde dormía habitualmente el/la niño/a hasta los 6 meses de vida?	☐ Cama compartida ☐ Cuna solo/a<br>☐ Otro: Especificar ____<br>☐ Omite ☐ Ns/Nc
**30.**	¿Con quién compartía la cama?	____<br>☐ Omite ☐ Ns/Nc
**31.**	¿En qué posición dormía habitualmente?	☐ Boca arriba<br>☐ Boca abajo<br>☐ De costado<br>☐ Omite ☐ Ns/Nc
**PERCEPCIONES DE LA MADRE**		II
**1.**	¿Tu hijo/a era como lo esperabas?	☐ Si ☐ No<br>☐ Omite ☐ Ns/Nc
**2.**	¿Te resultó fácil su crianza?	☐ Si ☐ No<br>☐ Omite ☐ Ns/Nc

FICHA	**MORTALIDAD INFANTIL DOMICILIARIA**<br>**Fundación Infant - Versión 2.1 - Octubre 2016**	**Florencio Varela** ☐<br>**Lomas de Zamora** ☐

**3.**	¿Cómo puede describir que era la salud de su niño/a en general?	☐ Omite ☐ Ns/Nc
**ALIMENTACION**		**III**
**1.**	Con respecto a la alimentación habitual de el/la niño/a , durante el primer año de vida, ¿Qué alimentos consumió?<br><br>Marcar todas las opciones que correspondan, desde su nacimiento.	Lactancia materna<br>☐ Si ☐ No ☐ Ns/Nc ☐ Omite<br>Leche complemento<br>☐ Si ☐ No ☐ Ns/Nc ☐ Omite<br>Otros líquidos<br>☐ Si ☐ No ☐ Ns/Nc ☐ Omite<br>Semisólidos/solidos<br>☐ Si ☐ No ☐ Ns/Nc ☐ Omite
**La siguiente pregunta corresponde solo a niños mayores de 5 meses**		
**2.**	¿Puede describir como era la alimentación de el/la niño/a en los últimos 3 meses de vida?	
**3.**	¿Dónde realizaba la mayor parte de las comidas?<br><br>Marcar todas las opciones posibles.	☐ Hogar ☐ Comedor comunitario<br>☐ Jardín/Escuela<br>☐ Otro: Especificar ____<br>☐ Omite ☐ Ns/Nc
**4.**	¿Qué comidas realizaba habitualmente en el día?<br><br>Marcar todas las opciones posibles.	☐ Desayuno ☐ Almuerzo<br>☐ Merienda ☐ Cena<br>☐ Omite ☐ Ns/Nc
**5.**	¿Consumía estos alimentos al menos una vez a la semana?<br><br>Marcar todas las opciones posibles.	☐ Verduras verdes ☐ Otras verduras<br>☐ Arroz, pastas ☐ Carnes rojas<br>☐ Pollo ☐ Pescado<br>☐ Frutas ☐ Agua segura<br>☐ Agua no segura ☐ Cereales, legumbres<br>☐ Huevos ☐ Lácteos(leche, crema, quesos, etc.)<br>☐ Aceite de cocina (Solo si utiliza aceite crudo, no frito)<br>☐ Otro: Especificar ____<br>☐ Omite ☐ Ns/Nc

FICHA	MORTALIDAD INFANTIL DOMICILIARIA<br>Fundación Infant - Versión 2.1 - Octubre 2016	Florencio Varela ☐<br>Lomas de Zamora ☐

CONTROLES DE SALUD — IV

1.	¿Lo/a llevaba a "control de niño/a sano/a"?	☐ Todos los meses ☐ A veces<br>☐ Nunca<br>☐ Omite ☐ Ns/Nc
	**(Si la respuesta es Ns/Nc, Nunca u Omite pasar a Pregunta 6)**	
2.	Lugar donde lo/a llevaba a hacer control:	☐ Hospital público ☐ Centro de salud<br>☐ Hospital privado ☐ Obra social<br>☐ En la casa<br>☐ Otro: Especificar ______<br>☐ Omite ☐ Ns/Nc
3.	En el caso de que asistiera a alguna Institución, escribir cual:	______
4.	Fecha de la primera consulta:	__ __ / __ __ / __ __ __ __<br>☐ Omite ☐ Ns/Nc
5.	Fecha de la última consulta:	__ __ / __ __ / __ __ __ __<br>☐ Omite ☐ Ns/Nc
6.	Si al control de niño/a sano/a lo llevó A veces o Nunca ¿Por qué lo cree posible?<br><br>Marcar todas las opciones posibles.	☐ No lo consideró necesario<br>☐ Obstáculos (temores, expectativas)<br>☐ No tenía acompañamiento para llevarlo<br>☐ No tenía con quien dejar a sus otros hijos<br>☐ Le quedaba muy lejos<br>☐ No tenía transporte desde el domicilio al servicio de salud<br>☐ No la atendían en el servicio de salud<br>☐ No había pediatra en el centro de salud y hospital<br>☐ Otro: Especificar ______<br>☐ Omite ☐ Ns/Nc
7.	¿Cuál fue el peso conocido del niño antes de fallecer?	__ __ __ __ __ gramos<br>☐ Omite ☐ Ns/Nc

VACUNAS — V

1.	¿Cómo era el estado del esquema de vacunas para la edad al fallecer, según el calendario nacional?	☐ Completas ☐ Incompletas<br>☐ Omite ☐ Ns/Nc

Identificación del Paciente: __ __ - __ __ - __ __ - __ __ __ - __ __

Codificacion: dos letras nombre, dos letras apellido y fecha de nacimiento

2. Si el esquema de vacunación estaba Incompleto para la edad al momento de fallecer ¿Por qué cree que sucedió?

Marcar todas las opciones posibles

- ☐ No lo consideró necesario
- ☐ Obstáculos (temores, expectativas)
- ☐ No tenía acompañamiento para llevarlo
- ☐ No tenía con quien dejar a sus otros hijos
- ☐ Le quedaba muy lejos
- ☐ No tenía transporte desde el domicilio al servicio de salud
- ☐ No la atendían en el servicio de salud
- ☐ No vacunaban en el centro de salud donde asistía
- ☐ El/la bebé estaba siempre enfermo/a y por eso me decían que no podía vacunarlo/a
- ☐ Otro: Especificar ____________
- ☐ Omite ☐ Ns/Nc

(Si tiene el carnet de vacunas, marque las vacunas administradas en el item 3)

3. Marque todas las vacunas que tiene documentadas en su carnet

SI tiene el carnet ☐ NO tiene el carnet ☐

Edad	Vacunas									
	BCG	Hepatiti B	Neumococo conjugada	Quintuple Pentavalente DTP-HB-Hib	Rotavirus	Cuadruple o Quintuple Pentavalente	Polio	Triple Viral SRP	Gripe	Hepatitis A HA	Varicela
Recien nacido	Unica dosis	Dosis neonatal									
2 meses			1º dosis	1º dosis	1º dosis		1º dosis				
4 meses			2º dosis	2º dosis	2º dosis		2º dosis				
6 meses				3º dosis			3º dosis				
12 meses			refuerzo					1º refuerzo		unica dosis	
15 meses									dosis anual		unica dosis
15-18 meses						1º refuerzo					
18 meses											
24 meses											

ANTECEDENTES DE ENFERMEDAD DE EL/LA NIÑO/A — VI

1. ¿Presentó alguna de estas enfermedades agudas en su vida?

Marcar todas las que tuvo.

- ☐ Ninguna ☐ Neumonía
- ☐ Bronquiolitis ☐ Otitis media aguda
- ☐ Otro: Especificar ____________
- ☐ Omite ☐ Ns/Nc

2. ¿Presentó alguna vez estos eventos?

Marcar todas las que tuvo.

- ☐ Caída de altura ☐ Fractura
- ☐ Quemadura ☐ Moretones en la piel
- ☐ Otro: Especificar ____________
- ☐ Omite ☐ Ns/Nc ☐ No

**3.**	¿El/la niño/a requirió internación/es? (excluyendo neonatología).	☐ Si, especificar ↓<br>[ ]<br>☐ No<br>☐ Omite ☐ Ns/Nc
**4.**	Marcar si presentaba alguna de las siguientes enfermedades (Comorbilidades) y enfermedades crònicas<br><br>Marcar o escribir todas las que tuvo.	☐ Atopía (rinitis alérgica, dermatitis atópica, eccema)<br>☐ Enfermedad respiratoria crónica<br>☐ Cardiopatía congénita<br>☐ Síndrome de Down<br>☐ Otro síndrome → [ ]<br>☐ Inmunodeficiencia<br>☐ [ ]<br>☐ Otra patología → [ ]<br>☐ Omite ☐ Ns/Nc ☐ No

ANTECEDENTES MATERNOS — C

ANTECEDENTES DE LA MADRE — I

**1.**	¿La mamá de el/la niño/a fallecido/a vive?	☐ Si ☐ No<br>☐ Omite ☐ Ns/Nc
**2.**	¿Vivía la mamá junto con su hijo/a antes de fallecer el/la niño/a?	☐ Si ☐ No<br>☐ Omite ☐ Ns/Nc
**3.**	¿Cuál es la fecha de nacimiento de la madre?	[ ][ ] / [ ][ ] / [ ][ ][ ][ ]<br>☐ Omite ☐ Ns/Nc
**4.**	¿Donde nació la madre?	Ciudad: [ ]<br>País: [ ]<br>☐ Omite ☐ Ns/Nc
**5.**	¿Cuál es el mayor nivel educativo alcanzado por la madre?	☐ Primaria incompleta<br>☐ Primaria completa<br>☐ Secundaria incompleta **(En curso)**<br>☐ Secundaria incompleta **(Abandonó)**<br>☐ Secundaria completa<br>☐ Terciario/Universitario incompleto<br>☐ Terciario/Universitario completo<br>☐ Omite ☐ Ns/Nc

FICHA	MORTALIDAD INFANTIL DOMICILIARIA<br>Fundación Infant - Versión 2.1 - Octubre 2016	Florencio Varela ☐<br>Lomas de Zamora ☐

**6.**	¿El padre la acompaño durante el embarazo?	☐ Si ☐ No<br>☐ Omite ☐ Ns/Nc
**7.**	Desde que nació el/la niño/a, ¿Cómo fue la relación con el padre del niño?	☐ Casados/pareja estable<br>☐ Separados con contacto frecuente<br>☐ Separados sin contacto<br>☐ Contacto conflictivo<br>☐ Otro: Especificar ____<br>☐ Omite ☐ Ns/Nc
**8.**	¿Donde vive la madre?	Barrio: ____<br>☐ Omite ☐ Ns/Nc
**9.**	¿Hace cuánto vive allí? (Marcar el tiempo correcto)	Cantidad de ☐☐ Meses ☐☐ Años<br>☐ Omite ☐ Ns/Nc
**10.**	¿Su vivienda anterior, era en este mismo barrio?	☐ Si ☐ No<br>☐ Omite ☐ Ns/Nc
**11.**	¿Cuántas veces cambio su vivienda en los últimos tres años?	☐ 0 ☐ 1 ☐ 2 ☐ 3<br>☐ más de 3 veces<br>☐ Omite ☐ Ns/Nc

ANTECEDENTES RESPIRATORIOS MATERNOS (II)

**1.**	¿Le ha diagnosticado a la madre asma o broncoespasmo un mèdico?	☐ Si ☐ No<br>☐ Omite ☐ Ns/Nc
**2.**	¿Presenta la madre rinitis alérgica u otra manifestación de alergia (en la piel, alergia a alimentos)?	☐ Si ☐ No<br>☐ Omite ☐ Ns/Nc

ANTECEDENTES PERINATOLÓGICOS MATERNOS (III)

**1.**	¿Cuántos embarazos tuvo la mamá en total?	☐☐<br>☐ Omite ☐ Ns/Nc
**2.**	¿Cuántos partos tuvo la mamá?	☐☐<br>☐ Omite ☐ Ns/Nc
**3.**	Cantidad de hijos vivos que tiene	☐☐<br>☐ Omite ☐ Ns/Nc

Identificación del Paciente: __ __ - __ __ - __ __ - __ __ __ - __ __

Codificacion: dos letras nombre, dos letras apellido y fecha de nacimiento

**4.**	¿Cuántos abortos tuvo la mama?	☐☐<br>☐ Omite ☐ Ns/Nc
**5.**	¿Cuántos hijos fallecido tiene la mama?	☐☐<br>☐ Omite ☐ Ns/Nc
**6.**	¿Tiene hijos que nacieron antes de tiempo?	☐ Si → Cuántos? ☐☐<br>Cuándo? ☐<br>☐ No<br>☐ Omite ☐ Ns/Nc
**7.**	¿Tiene hijos que nacieron con peso menor a 2500 gramos?	☐ Si → Cuántos? ☐☐<br>☐ No<br>☐ Omite ☐ Ns/Nc
**8.**	Vacunas que la mamá recibió durante el embarazo, completar según la documentación del control del embarazo	(see table below)
**9.**	¿Tuvo alguna enfermedad o complicación durante el embarazo?	☐ Si, cuáles → ☐ Fiebre<br>☐ Presión alta<br>☐ Azúcar alta/diabetes<br>☐ Anemia<br>☐ Otro ↓<br>☐<br>☐ No<br>☐ Omite ☐ Ns/Nc
**10.**	¿Cuántas veces durante el embarazo la madre consultó para control del embarazo de el/la niño/a?	☐ Nunca<br>☐ Menos de 1 vez por trimestre<br>☐ 1 vez o más por trimestre pero menos de 1 vez por mes<br>☐ 1 vez por mes o más<br>☐ Omite ☐ Ns/Nc

Vacuna	Si	No	Ns/Nc	Fecha aplicación
Antigripal	☐	☐	☐	☐
V.Acelular (DPaT)	☐	☐	☐	☐
Doble (DPT)	☐	☐	☐	☐

(si contesta Nunca, NS/Nc, Omite pasar a Preg. 15)

FICHA	MORTALIDAD INFANTIL DOMICILIARIA<br>Fundación Infant - Versión 2.1 - Octubre 2016	Florencio Varela ☐<br>Lomas de Zamora ☐

**11.**	¿En qué consistió el control?	☐ Control médico y ecográfico<br>☐ Control sólo médico<br>☐ Control sólo ecográfico<br>☐ Omite ☐ Ns/Nc
**12.**	¿Dónde realizó los controles durante el embarazo?<br>Marcar todas las opciones posibles.	☐ Hospital público ☐ Centro de salud<br>☐ Hospital privado ☐ Obra social<br>☐ En la casa<br>☐ Otro: Especificar ____<br>☐ Omite ☐ Ns/Nc
**13.**	En caso de haber nacido en una institución, escribir el nombre:	____
**14.**	¿Quién le realizó los controles del embarazo?<br>Marcar todas las opciones posibles.	☐ Medico ☐ Partera<br>☐ Enfermera<br>☐ Otro: Especificar ____<br>☐ Omite ☐ Ns/Nc
**15.**	Si no controló el embarazo, o fue menos de una vez por trimestre, ¿cuál cree que fueron los motivos?<br>Marcar todas las opciones posibles	☐ No lo consideró necesario<br>☐ Obstáculos (temores, expectativas)<br>☐ No tenía acompañamiento para llevarlo<br>☐ No tenía con quien dejar a sus otros hijos<br>☐ Le quedaba muy lejos<br>☐ No tenía transporte desde el domicilio al servicio de salud<br>☐ No la atendían en el servicio de salud<br>☐ No sabia que estaba embarazada<br>☐ Otro : Especificar ____<br>☐ Omite ☐ Ns/Nc
**16.**	Durante el embarazo, en alguna institución de salud o centro comunitario ¿le contaron acerca del embarazo, cuidados del recién nacido, etc.?	☐ Si ☐ No<br>☐ Omite ☐ Ns/Nc
**17.**	¿Sintió que tuvo dónde acudir para preguntar acerca de estos temas?	☐ Si ☐ No<br>☐ Omite ☐ Ns/Nc

Identificación del Paciente: __ __ - __ __ - __ __ - __ __ __ - __ __

**18.**	¿Trabajó y/o estudió durante el embarazo?	☐ Si ☐ No<br>☐ Omite ☐ Ns/Nc
**(si es NO, Omite, Ns/Nc pasar a " Habitos durante el embarazo")**		
**19.**	¿Que actividad desarrollaba?	☐ Empleada (administrativa / en comercio)<br>☐ Fábrica (operaria)<br>☐ Doméstica / portería<br>☐ Taller de costura<br>☐ Jubilada<br>☐ Desocupada (busca trabajo)<br>☐ Otro: Especificar ______<br>☐ Omite ☐ Ns/Nc
**20.**	¿Hasta que mes del embarazo trabajaba y /o estudiaba ?	☐ mes<br>☐ Omite ☐ Ns/Nc
**21.**	¿Cuántos días por semana trabajada y/o estudiaba?	☐ días<br>☐ Omite ☐ Ns/Nc
**22.**	¿Cuantas horas por día trabajaba y/o estudiaba ?	☐☐ horas<br>☐ Omite ☐ Ns/Nc
**HÁBITOS DURANTE EL EMBARAZO**		**IV**
**1.**	¿Fumó tabaco durante el embarazo?	☐ Si ☐ No<br>☐ Omite ☐ Ns/Nc
**(Si no fumó tabaco durante el embarazo, Ns/Nc u Omite, pasar al ítem 3)**		
**2.**	¿Durante que trimestre/s?	☐ 1° trimestre<br>☐ 2° trimestre<br>☐ 3° trimestre<br>☐ Omite ☐ Ns/Nc
**3.**	¿Consumió otras sustancias durante el embarazo? (incluye marihuana, alcohol, y otras drogas)	☐ Si ☐ No<br>☐ Omite ☐ Ns/Nc
**(Si contesta No, Ns/Nc u omite pasar a la pregunta 6)**		
**4.**	Especificar que otras sustancias consumió durante el embarazo	______

5.	¿Durante que trimestre/s?	☐ 1° trimestre<br>☐ 2° trimestre<br>☐ 3° trimestre<br>☐ Omite ☐ Ns/Nc
6.	¿Consumió algún medicamento durante el embarazo?	☐ Si ☐ No<br>☐ Omite ☐ Ns/Nc
**(Si contesta No, Ns/Nc u omite pasar a " Atecedentes Paternos")**		
7.	Especificar que medicamentos consumió durante el embarazo	
8.	¿Durante que trimestre/s?	☐ 1° trimestre<br>☐ 2° trimestre<br>☐ 3° trimestre<br>☐ Omite ☐ Ns/Nc

ANTECEDENTES PATERNOS — D

ANTECEDENTES DEL PADRE — I

1.	¿El padre de el/la niño/a fallecido/a vive?	☐ Si ☐ No<br>☐ Omite ☐ Ns/Nc
2.	¿Vivía el papá junto con su hijo/a antes de fallecer el/la niño/a?	☐ Si ☐ No<br>☐ Omite ☐ Ns/Nc
3.	¿Cuál es la fecha de nacimiento del padre?	☐☐ / ☐☐ / ☐☐☐☐<br>☐ Omite ☐ Ns/Nc
4.	¿Donde nació el padre?	Ciudad: ____<br>País: ____<br>☐ Omite ☐ Ns/Nc
5.	¿Cuál es el mayor nivel educativo alcanzado por el padre?	☐ Primaria incompleta<br>☐ Primaria completa<br>☐ Secundaria incompleta **(En curso)**<br>☐ Secundaria incompleta **(Abandonó)**<br>☐ Secundaria completa<br>☐ Terciario/Universitario incompleto<br>☐ Terciario/Universitario completo<br>☐ Omite ☐ Ns/Nc

FICHA	MORTALIDAD INFANTIL DOMICILIARIA<br>Fundación Infant - Versión 2.1 - Octubre 2016	Florencio Varela ☐<br>Lomas de Zamora ☐

**6.**	¿Vive con la mamá del niño/a?	☐ Si ☐ No<br>☐ Omite ☐ Ns/Nc
**(Si contesta Si pasar a " Atecedentes Respiratorios Paternos")**		
**7.**	¿Donde vive el padre?	Barrio: ☐<br>☐ Omite ☐ Ns/Nc
**8.**	¿Hace cuánto vive allí? (Escribir el tiempo correcto)	Cantidad de ☐☐ Meses ☐☐ Años<br>☐ Omite ☐ Ns/Nc
**9.**	¿Tiene costumbre de cambiar de casa?	☐ Si → Motivo ☐<br>☐ No<br>☐ Omite ☐ Ns/Nc
**ANTECEDENTES RESPIRATORIOS PATERNOS**		**II**
**1.**	¿Le ha diagnosticado asma o broncoespasmo un médico?	☐ Si ☐ No<br>☐ Omite ☐ Ns/Nc
**2.**	¿Presenta rinitis alérgica u otra manifestación de alergia (en la piel, alergia a alimentos)?	☐ Si ☐ No<br>☐ Omite ☐ Ns/Nc
**ANTECEDENTES HERMANOS**		**E**
**ANTECEDENTES DE HERMANOS**		**I**
**1.**	El niño fallecido, tiene hermanos de diferente madre y/o padre	☐ Si ☐ No<br>☐ Omite ☐ Ns/Nc
**ANTECEDENTES RESPIRATORIOS DE HERMANOS**		**II**
**1.**	¿Un médico le ha diagnosticado a los hermanos asma o broncoespasmo?	☐ Si ☐ No<br>☐ Omite ☐ Ns/Nc
**2.**	¿Presentan los hermanos rinitis alérgica u otra manifestación de alergia (en la piel, alergia a alimentos)?	☐ Si ☐ No<br>☐ Omite ☐ Ns/Nc
**REDES Y COMUNIDAD**		**F**
**CONVIVIENTES**		**I**
**1.**	¿Cuántas personas conviven juntas en la misma casa que el niño/a fallecida? Especificar número excluyendo a el niño/a fallecido/a?	☐☐ personas<br>☐ Omite ☐ Ns/Nc

FICHA	MORTALIDAD INFANTIL DOMICILIARIA<br>Fundación Infant - Versión 2.1 - Octubre 2016	Florencio Varela ☐<br>Lomas de Zamora ☐

2.	¿Quiénes componen el grupo de personas convivientes?<br>(Parentescos en relación al niño/a)<br><br>Marcar todas las opciones posibles	☐ Madre<br>☐ Padre<br>☐ Tío/a<br>☐ Hermano/a<br>☐ Abuelo/a	☐ No familiar<br>☐ Otro menor de 12 años<br>☐ Otro mayor de 12 años<br>☐ Omite<br>☐ Ns/Nc
3.	Quienes conforman la red social primaria de convivientes	☐ Madre<br>☐ Padre	☐ Pareja de la Madre<br>☐ Pareja del Padre
4.	Personas con las cuales la madre/cuidador comparte preocupaciones sobre la salud de los niños.<br><br>Marcar todas las opciones posibles	☐ Madre<br>☐ Padre<br>☐ Tío/a<br>☐ Hermano/a<br>☐ Abuelo/a	☐ No familiar<br>☐ Otro menor de 12 años<br>☐ Otro mayor de 12 años<br>☐ Omite<br>☐ Ns/Nc
5.	¿Alguna otra persona participó / colaboró en la crianza de el/la niño/a?	☐ Si<br>☐ Omite	☐ No<br>☐ Ns/Nc
6.	Detallar que persona participó / colaboró en la crianza de el/la niño/a.<br><br>Marcar todas las opciones posibles	☐ Madre<br>☐ Padre<br>☐ Tío/a<br>☐ Hermano/a<br>☐ Abuelo/a	☐ No familiar<br>☐ Otro menor de 12 años<br>☐ Otro mayor de 12 años<br>☐ Omite<br>☐ Ns/Nc
7.	¿Hay convivientes que hayan estado con alguna enfermedad en la última semana previa al fallecimiento de el/la niño/a?	☐ Si<br>☐ Omite	☐ No<br>☐ Ns/Nc
**(Si no tuvieron enfermedades, Omite, Ns/Nc pasar a pregunta 9)**			
8.	¿Cuál fue/fueron esa/s enfermedad/es?, Especificar:	________<br>☐ Omite	☐ Ns/Nc
9.	¿Hubo alguna situación estresante o evento grave en las últimas semanas, antes de fallecer, que haya afectado a uno o más convivientes?	☐ Si<br>☐ Omite	☐ No<br>☐ Ns/Nc
**(Si no presentaron situaciones estresantes pasar a Item "Fuentes de ingresos económicos en convivientes")**			
10.	¿Cuál fue/fueron esa/s situación/es?, Especificar:	________<br>☐ Omite	☐ Ns/Nc

Identificación del Paciente: __ __ - __ __ - __ __ - __ __ __ - __ __

Codificacion: dos letras nombre, dos letras apellido y fecha de nacimiento

FICHA	**MORTALIDAD INFANTIL DOMICILIARIA**<br>**Fundación Infant - Versión 2.1 - Octubre 2016**	**Florencio Varela** ☐<br>**Lomas de Zamora** ☐

**FUENTES DE INGRESOS ECONÓMICOS EN CONVIVIENTES**		**II**
**1.**	¿Cuántos miembros convivientes trabajan?	☐☐ personas<br>☐ Omite ☐ Ns/Nc
**2.**	¿Que tipo de trabajo realizan?<br>Marcar todas las opciones	☐ Regularizado<br>☐ Informal<br>☐ Otro<br>☐ Omite ☐ Ns/Nc
**3.**	¿La madre recibe apoyo económico de otras personas que no sean convivientes?	☐ Si ☐ No<br>☐ Omite ☐ Ns/Nc
**EDUCACIÓN DE LOS CONVIVIENTES**		**III**
**1.**	¿Cuál es el máximo nivel educativo alcanzado en el grupo conviviente?	☐ Primaria incompleta<br>☐ Primaria completa<br>☐ Secundaria incompleta **(En curso)**<br>☐ Secundaria incompleta **(Anbandonó)**<br>☐ Secundaria completa<br>☐ Terciario/Universitario incompleto<br>☐ Terciario/Universitario completo<br>☐ Omite ☐ Ns/Nc
**HÁBITOS DE LOS CONVIVIENTES**		**IV**
**1.**	¿Fumaba alguno de los convivientes mientras vivía el/la niño/a?	☐ Si ☐ No<br>☐ Omite ☐ Ns/Nc
**2.**	¿Consumía alguno de los convivientes otra droga/sustancia mientras el/la niño/a vivía? (droga, alcohol)	☐ Si ☐ No<br>☐ Omite ☐ Ns/Nc
**REDES Y COMUNIDAD (EN RELACIÓN A LA MADRE / CUIDADOR)**		**V**
**1.**	¿Existen situaciones de violencia en la red primaria?(vínculos violentos,descuido,maltrato,consumo,conflictos con la ley etc.)?	☐ Si ☐ No<br>☐ Omite ☐ Ns/Nc
**2.**	¿Conoce algún grupo comunitario, religioso, de actividades o encuentros barriales, recreativos, culturales, o políticos?	☐ Si ☐ No<br>☐ Omite ☐ Ns/Nc
**(Si No Conoce, Omite, Ns/Nc pasar a pregunta 5)**		

3.	¿Qué grupo/s conoce?, Especificar:	
4.	Si participa en alguno de esos grupos, especificar cual/es:	☐ Omite ☐ Ns/Nc
5.	¿Realiza alguna actividad de recreación?	☐ Si ☐ No ☐ Omite ☐ Ns/Nc
6.	¿Tiene amigas/os?	☐ Si ☐ No ☐ Omite ☐ Ns/Nc
7.	Ante un problema afectivo, económico, etc., si necesitas pedir un favor, ¿a quien se lo pedís?	☐ Omite ☐ Ns/Nc
8.	¿La madre o alguno de los convivientes recibe apoyo económico del Estado? (Asignación universal, bolsones, pensiones, etc.)	☐ Si ☐ No ☐ Omite ☐ Ns/Nc

SOCIABILIZACIÓN Y MEDIO AMBIENTE — G

JARDÍN DE INFANTES O MATERNAL — I

1.	¿Conoce algún jardín de infantes o maternal o salita donde pueda dejar a los niños para su cuidado?	☐ Si ☐ No ☐ Omite ☐ Ns/Nc
	**(Si es NO, Omite, Ns/Nc pasar a Item " Juegos")**	
2.	¿Cuáles/es es/son es/esos lugar/es?	
3	¿A que lugar/es concurría habitualmente?	Nombre:<br>Barrio:<br>Otro:

JUEGO — II

1.	¿Jugaba el/la niño/a?	☐ Si ☐ No ☐ Omite ☐ Ns/Nc
2.	¿A que jugaba?	

CUIDADOS PERSONALES — III

1.	Describir los hábitos de cuidado personal que el/la niño/a tenía, siguiendo la siguiente guía: higiene corporal, uso de jabón / shampoo, lavado de manos habitual, cepillado de dientes diario, ropa limpia, utensilios de cocina limpios, etc.	☐ Omite ☐ Ns/Nc

FICHA	MORTALIDAD INFANTIL DOMICILIARIA<br>Fundación Infant - Versión 2.1 - Octubre 2016	Florencio Varela ☐<br>Lomas de Zamora ☐

AMBIENTE SEGURO - PREVENCIÓN DE ACCIDENTES

**1.**	Marcar todas las situaciones de riesgo de accidentes que se encuentran en la casa	☐ Enchufes sin protecciòn /Art. Eléc. Descuidados<br>☐ Riesgo de quemaduras por agua caliente / estufas / etc.<br>☐ Riesgo de ahogamiento / sumersión en baño, tachos, etc.<br>☐ Riesgo de caída de altura (camas elevadas/ escaleras/ casa en altura/ etc.)<br>☐ Piedras y vidrios en el piso de la casa y en el exterior<br>☐ Puerta de acceso a la vivienda sin cerradura<br>☐ Animales en la casa no adiestrados / no vacunados<br>☐ Elementos de limpieza / medicamentos u otros tóxicos a la alcance de los niños.<br>☐ Otro, especificar↓<br>________<br>☐ Omite ☐ Ns/Nc

VIVIENDA Y SERVICIOS (VIVIENDA DONDE RESIDÍA EL NIÑO/A) H

VIVIENDA - DATOS OBTENIDOS POR OBSERVACIÓN I

**1.**	Municipio de la vivienda:	________
**2.**	Tipo de zona donde vive:	☐ Urbana<br>☐ Rural<br>☐ Asentamiento
**3.**	Condiciones mayoritarias de las calles del barrio:	☐ Tierra<br>☐ Asfalto<br>☐ Tosca
**4.**	Zona anegable:	☐ Si ☐ No<br>☐ Omite ☐ Ns/Nc
**5.**	Existencia de sitios contaminantes (basurales, criaderos, curtiembres etc.)	☐ Si ☐ No<br>☐ Omite ☐ Ns/Nc
**6.**	Acceso a medios de transporte público, conectividades.	☐ Fácil acceso<br>☐ Difícil acceso<br>☐ Otro
**7.**	Número de habitaciones (excluyendo baño y cocina):	☐☐ habitaciones<br>☐ Omite ☐ Ns/Nc

FICHA	MORTALIDAD INFANTIL DOMICILIARIA<br>Fundación Infant - Versión 2.1 - Octubre 2016	Florencio Varela ☐<br>Lomas de Zamora ☐

**8.**	La vivienda del niño/a era ¿independiente o múltiple?:	☐ Independiente<br>☐ Omite	☐ Múltiple<br>☐ Ns/Nc
**9.**	Materiales principales de la vivienda:	☐ Chapa<br>☐ Cartón<br>☐ Cemento	☐ Madera<br>☐ Adobe<br>☐ Otros
**10.**	Techo:	☐ Chapa<br>☐ Cartón	☐ Losa<br>☐ Otros
**11.**	Piso:	☐ Tierra<br>☐ Cemento / mosaico	☐ Cemento<br>☐ Otros
**12.**	Baño: Marcar todas las que correspondan	☐ Único familiar<br>☐ Fuera de la casa<br>☐ Inodoro<br>☐ Omite	☐ Compartido<br>☐ Dentro de la casa<br>☐ Letrina<br>☐ Ns/Nc
**SERVICIOS - DATOS OBTENIDOS POR OBSERVACIÓN**			II
**1.**	Agua de consumo:	☐ Segura<br>☐ Omite	☐ No segura<br>☐ Ns/Nc
**2.**	Lugar de acceso al agua :	☐ Dentro de la vivienda<br>☐ Omite	☐ Fuera de la vivienda<br>☐ Ns/Nc
**3.**	Fuente del agua:	☐ Corriente<br>☐ Otro Especificar<br>☐ Omite	☐ De pozo<br>________<br>☐ Ns/Nc
**4.**	Modo de calefacción:	☐ Gas natural<br>☐ Leña<br>☐ Omite	☐ Garrafa<br>☐ Eléctrica<br>☐ Ns/Nc
**5.**	Modo de cocina:	☐ Gas natural<br>☐ Leña<br>☐ Omite	☐ Garrafa<br>☐ Eléctrica<br>☐ Ns/Nc
**6.**	Describir elementos de la vivienda, por fuera y por dentro, que le llamen la atención y que no estén especificadas en otra parte:		

Identificación del Paciente: __ __ - __ __ - __ __ - __ __ __ - __ __

Codificacion: dos letras nombre, dos letras apellido y fecha de nacimiento

FICHA	**MORTALIDAD INFANTIL DOMICILIARIA**<br>**Fundación Infant - Versión 2.1 - Octubre 2016**	**Florencio Varela** ☐<br>**Lomas de Zamora** ☐

**REGISTROS MÉDICOS (Libreta sanitaria- Historia Clínica - Resumen Historia Clínica)**			**I**
**(Si No posee registros médicos pasar a " Proceso de Muerte")**			
**EMBARAZO**			**I**
**1.**	¿Posee la madre/familia la libreta del control del embarazo?	☐ Si<br>☐ Omite	☐ No<br>☐ Ns/Nc
**2.**	Datos que la libreta del embarazo posee:	☐ Completa<br>☐ Omite	☐ Incompleta<br>☐ Ns/Nc
**CONTROL DEL NIÑO/A**			**II**
**1.**	¿Tiene la libreta de control de salud de el/la niño/a?	☐ Si<br>☐ Omite	☐ No<br>☐ Ns/Nc
**2.**	Datos que la libreta del control de salud posee:	☐ Completa<br>☐ Omite	☐ Incompleta<br>☐ Ns/Nc
**NEONATOLOGÍA**			**III**
**1.**	¿Existe historia clínica del niño/a en neonatología?	☐ Si<br>☐ Omite	☐ No<br>☐ Ns/Nc
**2.**	Datos que la historia clínica de neonatología posee:	☐ Completa<br>☐ Omite	☐ Incompleta<br>☐ Ns/Nc
**HISTORIA CLÍNICA HOSPITAL**			**IV**
**1.**	¿Existe historia clínica del niño/a en algún hospital?	☐ Si<br>☐ Omite	☐ No<br>☐ Ns/Nc
**2.**	Datos que la historia clínica del hospital posee:	☐ Completa<br>☐ Omite	☐ Incompleta<br>☐ Ns/Nc
**HISTORIA CLÍNICA CENTRO DE SALUD**			**V**
**1.**	¿Existe historia clínica del niño/a en un centro de salud?	☐ Si<br>☐ Omite	☐ No<br>☐ Ns/Nc
**2.**	Datos que la historia clínica del centro de salud posee:	☐ Completa<br>☐ Omite	☐ Incompleta<br>☐ Ns/Nc
**PROCESO DEL EVENTO DE MUERTE**			**J**
**TIEMPO Y LUGAR DE MUERTE**			**I**
**1.**	¿Cuál es la fecha del fallecimiento del niño/niña ?	☐☐ / ☐☐ / ☐☐☐☐	

Identificación del Paciente: __ __ - __ __ - __ __ - __ __ __ - __ __

Codificacion: dos letras nombre, dos letras apellido y fecha de nacimiento

2.	¿Cuánto tiempo duró la enfermedad que le provocó la muerte?	☐☐ días ☐☐ meses<br>☐ Omite ☐ Ns/Nc
3.	¿Dónde falleció el niño/niña?	☐ Hospital ☐ Centro de salud<br>☐ Camino al hospital o centro de salud<br>☐ Hogar<br>☐ Otro: Especificar ____<br>☐ Omite ☐ Ns/Nc
**AUTOPSIA**		**II**
1.	¿Tiene autopsia realizada?	☐ Si ☐ No<br>☐ Omite ☐ Ns/Nc
**SISTEMA DE SALUD Y ACCESIBILIDAD EN RELACION AL EVENTO**		**III**
1.	¿Concurrió a algún lugar de salud durante la enfermedad que tuvo el/la niño/a ?	☐ Si ☐ No<br>☐ Omite ☐ Ns/Nc
**(Si Responde NO, Ns/Nc u Omite pasar a pregunta 11)**		
2.	¿A qué lugar de salud o servicio concurrió?	☐ Hospital público ☐ Centro de salud<br>☐ Hospital privado ☐ Obra social<br>☐ En la casa<br>☐ Otro: Especificar ____<br>☐ Omite ☐ Ns/Nc
3.	¿Fue atendido por el mismo médico/personal del equipo de salud que lo veía habitualmente?	☐ Si ☐ No<br>☐ Omite ☐ Ns/Nc
4.	¿Cuánto tiempo de viaje tuvo hasta el lugar donde fue asistido?	☐☐ minutos ☐☐ horas<br>☐ Omite ☐ Ns/Nc
5.	¿La atendieron con turno?	☐ Si ☐ No<br>☐ Omite ☐ Ns/Nc
6.	¿Cuándo pidió el turno, accedió fácilmente?	☐ Si ☐ No<br>☐ Omite ☐ Ns/Nc
7.	Entre que llegó y el niño/niña fue atendido, ¿cuánto tiempo pasó?	☐ Poco tiempo ☐ Mucho tiempo<br>☐ Omite ☐ Ns/Nc
8.	¿Le pidieron que realice algún estudio médico?	☐ Si ☐ No<br>☐ Omite ☐ Ns/Nc
**(Si es NO, Omite, Ns/Nc pasar a" Representacion Grafica del proceso de Muerte")**		

FICHA	MORTALIDAD INFANTIL DOMICILIARIA<br>Fundación Infant - Versión 2.1 - Octubre 2016	Florencio Varela ☐<br>Lomas de Zamora ☐

**9.**	¿Se hizo los estudios que le pidieron?	☐ Si ☐ No<br>☐ Omite ☐ Ns/Nc
**10.**	¿Cuál/es son los estudios que le realizaron al niño/a ? Describir	☐ Omite ☐ Ns/Nc
**11.**	Si no concurrió a algún centro de salud, ¿Cuál fue la razón por lo que no concurrió?<br><br>Marque todas las opciones posibles	☐ El niño/niña estaba bien, no necesitaba control médico<br>☐ Obstáculos (temores, expectativas)<br>☐ No tiene acompañamiento<br>☐ No tiene con quien dejar a sus hijos<br>☐ Le queda muy lejos<br>☐ No tiene transporte desde el domicilio al servicio de salud<br>☐ No la atienden en el servicio de salud<br>☐ Inseguridad<br>☐ No hay turnos ☐ No hay médicos<br>☐ Omite ☐ Ns/Nc

REPRESENTACIÓN GRAFICA DEL PROCESO **IV**

**1.**	**Lugar del fallecimiento(1)** ☐ →	Hora fallecido: __ __ : __ __<br>☐ Auto particular ☐ Transporte publico<br>☐ A pie ☐ Ambulancia<br>☐ Patrullero
	↓<br>Destino(2): ☐ →	Hora de llegada: __ __ : __ __<br>☐ A pie ☐ Ambulancia<br>☐ Patrullero<br>☐ Auto particular ☐ Transporte publico
	↓<br>Destino(2): ☐ →	Hora de llegada: __ __ : __ __<br>☐ A pie ☐ Ambulancia<br>☐ Patrullero<br>☐ Auto particular ☐ Transporte publico
	↓<br>Destino(2): ☐ →	Hora de llegada: __ __ : __ __

(1) Lugar del fallecimiento:

A	Hogar
B	Institución
C	Medio de transporte
D	Otro (especificar en el recuadro)

(2) Destinos:

1	Centro de salud
2	Otro domicilio particular
3	Hospital - guardia externa
4	Hospital - Morgue
5	Morgue judicial
6	Otro (especificar en el recuadro)

Identificación del Paciente: __ __ - __ __ - __ __ - __ __ __ - __ __

Codificacion: dos letras nombre, dos letras apellido y fecha de nacimiento

FICHA	MORTALIDAD INFANTIL DOMICILIARIA<br>Fundación Infant - Versión 2.1 - Octubre 2016	Florencio Varela ☐<br>Lomas de Zamora ☐

EVENTO DE MUERTE K

ANTECEDENTES NEONATALES PREVIOS AL EVENTO DE MUERTE I

**1.**	¿Tenía el/la niño/a al nacer algún moretón o marcas de lastimaduras en su cuerpo?	☐ Si ☐ No<br>☐ Omite ☐ Ns/Nc
**2.**	Al nacer ¿Tenía el/la niño/a alguna parte diferente o anormalidad en su cuerpo? Por ejemplo, alguna parte del cuerpo muy grande o muy chica.	☐ Si ☐ No<br>☐ Omite ☐ Ns/Nc
**3.**	¿Donde tenía esas anormalidades al momento de su nacimiento?	☐ La cabeza era muy chiquita<br>☐ La cabeza era muy grande<br>☐ Tenía un tumor o una masa en la parte de atrás de la cabeza o en la columna<br>☐ Otro: Especificar ________<br>☐ Omite ☐ Ns/Nc
**4.**	¿Respiró el/la niño/a inmediatamente despúes de nacer?	☐ Si ☐ No<br>☐ Omite ☐ Ns/Nc
**5.**	¿Tuvo el/la niño/a al nacer dificultades para respirar?	☐ Si ☐ No<br>☐ Omite ☐ Ns/Nc
**6.**	¿Se hizo algo en el momento que nació para ayudarlo/a que pueda respirar?	☐ Si ☐ No<br>☐ Omite ☐ Ns/Nc
**7.**	¿Lloró el/la niño/a inmediatamente después de nacer?	☐ Si ☐ No<br>☐ Omite ☐ Ns/Nc
**8.**	¿Cuánto tiempo tardó el/la niño/a en llorar despúes de nacer?<br>Marcar una sola opción.	☐ En los primeros 5 minutos de nacer<br>☐ En los primeros 6-30 minutos de nacer<br>☐ Nunca<br>☐ Omite ☐ Ns/Nc
**9.**	¿En algún momento después del parto, dejó de ser capaz de llorar?	☐ Si ☐ No<br>☐ Omite ☐ Ns/Nc
**10.**	¿Cuánto tiempo antes de fallecer el/la niño/a dejo de llorar?	☐ Menos de 1 dia ☐ 1 dia o más<br>☐ Omite ☐ Ns/Nc
**11.**	¿El/la bebé fue capaz de succionar bien en primer día de vida?	☐ Si ☐ No<br>☐ Omite ☐ Ns/Nc

Identificación del Paciente: __ __ - __ __ - __ __ - __ __ __ - __ __

Codificacion: dos letras nombre, dos letras apellido y fecha de nacimiento

**12.**	¿El/la niño/a pudo succionar bien en algún momento?	☐ Si<br>☐ Omite	☐ No<br>☐ Ns/Nc
**13.**	¿Alguna vez dejó de ser capaz de succionar normalmente?	☐ Si<br>☐ Omite	☐ No<br>☐ Ns/Nc
**14.**	¿Cuánto tiempo después de nacer el/la niño/a dejó de ser capaz de succionar?	☐ Menos de 1 dia<br>☐ Omite	☐ 1 dia o más<br>☐ Ns/Nc
**15.**	¿Alguna vez dejó de ser capaz de succionar normalmente?	☐ Menos de 1 dia<br>☐ Omite	☐ 1 dia o más<br>☐ Ns/Nc
**16.**	Cuándo dejó de poder succionar normalmente,¿podía abrir la boca?	☐ Si<br>☐ Omite	☐ No<br>☐ Ns/Nc
**EVENTO DE MUERTE**			**II**
**1.**	Durante el tiempo que tuvo la enfermedad que le provocó la muerte ¿tuvo dificultad para respirar normalmente?	☐ Si<br>☐ Omite	☐ No<br>☐ Ns/Nc
	**(Si responde No,Omite, Ns/Nc pasar a Preg 3)**		
**2.**	¿Cuántos días duró la dificultad respiratoria?	☐☐ días<br>☐ Omite	☐ Ns/Nc
**3.**	Durante el tiempo que tuvo la enfermedad que le provocó la muerte ¿El /la niño/a tuvo tos?	☐ Si<br>☐ Omite	☐ No<br>☐ Ns/Nc
	**(Si responde No,Omite, Ns/Nc pasar a Preg 7)**		
**4.**	¿Cuántos dias duró la tos?	☐☐ días<br>☐ Omite	☐ Ns/Nc
**5.**	¿La tos era muy grave, intensa? ¿Cambió de color la cara o boca, se puso morado/a azul?	☐ Si<br>☐ Omite	☐ No<br>☐ Ns/Nc
**6.**	¿El/la niño/a vomitó después de toser?	☐ Si<br>☐ Omite	☐ No<br>☐ Ns/Nc
**7.**	Durante la enfermedad que le provocó la muerte ¿estuvo respirando más rápido de lo habitual?	☐ Si<br>☐ Omite	☐ No<br>☐ Ns/Nc
**8.**	¿Cuántos días duró la respiración más rápida?	☐☐ días<br>☐ Omite	☐ Ns/Nc
**9.**	Durante el tiempo que tuvo la enfermedad que le provocó la muerte ¿se le hundía el pecho cuando respiraba?	☐ Si<br>☐ Omite	☐ No<br>☐ Ns/Nc

**10.**	Durante el tiempo que tuvo la enfermedad que le provocó la muerte ¿hacia ruido de quejidos?	☐ Si<br>☐ Omite	☐ No<br>☐ Ns/Nc
**11.**	Durante el tiempo que tuvo la enfermedad que le provocó la muerte ¿tuvo espasmos o convulsiones?	☐ Si<br>☐ Omite	☐ No<br>☐ Ns/Nc
**12.**	Durante el tiempo que tuvo la enfermedad que le provocó la muerte ¿El/la niño/a tuvo convulsiones generalizadas o localizadas?	☐ Si<br>☐ Omite	☐ No<br>☐ Ns/Nc
**13.**	¿Durante el tiempo que tuvo la enfermedad que le provocó la muerte ¿Tuvo el/la niño/a rigidez en el cuello?	☐ Si<br>☐ Omite	☐ No<br>☐ Ns/Nc
**14.**	Durante el tiempo que tuvo la enfermedad que le provocó la muerte ¿tuvo la mollerita/fontanela abultada, hinchada?	☐ Si<br>☐ Omite	☐ No<br>☐ Ns/Nc
**15.**	Durante el tiempo que tuvo la enfermedad que le provocó la muerte ¿El/la niño/a tuvo fiebre?	☐ Si<br>☐ Omite	☐ No<br>☐ Ns/Nc
	**(Si responde No,Omite, Ns/Nc pasar a Preg 18)**		
**16.**	¿Cuántos días duró la fiebre?	☐☐ días<br>☐ Omite	☐ Ns/Nc
**17.**	¿Como fue la gravedad de la fiebre?	☐ Leve (<38ºC)<br>☐ Severa (>39ºC, o con cambios de color en piel)<br>☐ Omite	☐ Moderada (entre 38-39ºC)<br>☐ Ns/Nc
**18.**	Durante el tiempo que tuvo la enfermedad que le provocó la muerte ¿lo/a notó frío/a cuando lo/a tocaba?	☐ Si<br>☐ Omite	☐ No<br>☐ Ns/Nc
	**(Si responde No,Omite, Ns/Nc pasar a Preg 20)**		
**19.**	¿Cuántos días lo/a sintió frio/a cuando lo/a tocaba?	☐☐ días<br>☐ Omite	☐ Ns/Nc
**20.**	Durante el tiempo que tuvo la enfermedad que le provocó la muerte ¿estuvo dormido, difícil de despertar luego de haber estado conectado/a y despierto/a?	☐ Si<br>☐ Omite	☐ No<br>☐ Ns/Nc
**21.**	Durante el tiempo que tuvo la enfermedad que le provocó la muerte ¿estuvo inconciente, indiferente?	☐ Si<br>☐ Omite	☐ No<br>☐ Ns/Nc
**22.**	Durante el tiempo que tuvo la enfermedad que le provocó la muerte ¿hacía el/la niño/a más veces caca de lo habitual, o era más líquida y/o la caca fue más abundante?	☐ Si<br>☐ Omite	☐ No<br>☐ Ns/Nc
	**(Si responde No,Omite, Ns/Nc pasar a Preg 27)**		

**23.**	¿Cuántas veces hizo caca el/la niño/a el día que hizo más cantidad de lo habitual?	☐☐ veces<br>☐ Omite ☐ Ns/Nc
**24.**	¿Cuántos días antes de fallecer comenzó a hacer más veces caca de lo habitual o las cacas comenzaron a ser mas liquidas de lo habitual?	☐☐ días ☐ Menos de 24 hs<br>☐ Omite ☐ Ns/Nc
**25.**	¿Las cacas más frecuentes de lo habitual o las cacas más líquidas de lo habitual continuaron hasta el día que el/la niño/a falleció?	☐ Si ☐ No<br>☐ Omite ☐ Ns/Nc
**26.**	¿Cuántos días antes de fallecer dejó de hacer cacas más líquidas de lo habitual o las cacas dejaron de ser más líquidas?	☐☐ días ☐ Menos de 24 hs<br>☐ Omite ☐ Ns/Nc
**27.**	¿En las cacas que hacía se veía sangre?	☐ Si ☐ No<br>☐ Omite ☐ Ns/Nc
**28.**	Durante el tiempo que tuvo la enfermedad que le provocó la muerte ¿el/la niño/a vomitaba todo?	☐ Si ☐ No<br>☐ Omite ☐ Ns/Nc
**29.**	Durante el tiempo que tuvo la enfermedad que le provocó la muerte, ¿tuvo erupción - rash en la piel?	☐ Si ☐ No<br>☐ Omite ☐ Ns/Nc
**30.**	Durante el tiempo que tuvo la enfermedad que le provocó la muerte, ¿tuvo ulceras, lesiones o lastimaduras en la piel??	☐ Si ☐ No<br>☐ Omite ☐ Ns/Nc
**31.**	¿Parecía que el/la niño/a estaba sano/a y luego murió de repente, súbitamente?	☐ Si ☐ No<br>☐ Omite ☐ Ns/Nc
**32.**	¿Sufrió el/la niño/a alguna de las siguientes heridas o accidentes?<br><br>Marcar todas en las que responda Si.	☐ Caída ☐ Accidente de transito<br>☐ Ahogamiento ☐ Intoxicación<br>☐ Quemadura ☐ Mordedura de animal<br>☐ Violencia (suicidio, homicidio, abuso, etc)<br>☐ Otro: Especificar ________<br>☐ Omite ☐ Ns/Nc ☐ No

(Si responde No,Omite, Ns/Nc pasar a "Antecedentes en la comunidad")

**33.**	¿La herida o accidente fueron intencionales, provocadas o Accidentales?	☐ Si ☐ No<br>☐ Omite ☐ Ns/Nc
**34.**	¿Cuántas horas sobrivivió después de sufrir la herida o accidente?	☐☐ horas ☐☐ dias<br>☐ Omite ☐ Ns/Nc

ANTECEDENTES EN LA COMUNIDAD — L

FALLECIDOS EN LA COMUNIDAD — I

**1.**	¿Existe algún caso de niño/a fallecido , menor a 14 años en los últimos 12 meses, en la familia, grupo conviviente o vecino cercano del/la niño/a fallecido/a?	☐ Si ☐ No<br>☐ Omite ☐ Ns/Nc
**2.**	¿Quién falleció?	☐ Conviviente ☐ Primo /Tio<br>☐ Vecino ☐ Amigo<br>☐ Otro: Especificar ____<br>☐ Omite ☐ Ns/Nc
**3.**	¿Cuándo falleció?	☐☐ Mes ☐☐☐☐ Año<br>☐ Omite ☐ Ns/Nc
**4.**	Causa del fallecimiento	☐ Accidente ☐ Enfermedad<br>☐ Omite ☐ Ns/Nc
**5.**	Lugar del fallecimiento	☐ Hogar ☐ Vía Pública<br>☐ Omite ☐ Ns/Nc
**6.**	Observaciones	

DATOS DEL FORMULARIO — Z

ENTREVISTADOR — I

**1.**	Nombre y apellido<br>Firma<br>Fecha	____<br>____<br>☐☐ / ☐☐ / ☐☐☐☐

FICHA	MORTALIDAD INFANTIL DOMICILIARIA<br>Fundación Infant - Versión 2.1 - Octubre 2016	Florencio Varela ☐<br>Lomas de Zamora ☐

2.	Nombre y apellido	
	Firma	
	Fecha	__ __ / __ __ / __ __ __ __
3.	Nombre y apellido	
	Firma	
	Fecha	__ __ / __ __ / __ __ __ __
**REVISIÓN**		II
1.	Nombre y apellido	
	Fecha	__ __ / __ __ / __ __ __ __
2.	Nombre y apellido	
	Fecha	__ __ / __ __ / __ __ __ __
3.	Nombre y apellido	
	Fecha	__ __ / __ __ / __ __ __ __
**CARGA EN BASE DE DATOS**		III
1.	Nombre y apellido	
	Fecha	__ __ / __ __ / __ __ __ __
2.	Nombre y apellido	
	Fecha	__ __ / __ __ / __ __ __ __
3.	Nombre y apellido	
	Fecha	__ __ / __ __ / __ __ __ __

Identificación del Paciente: __ __ - __ __ - __ __ - __ __ __ - __ __

Codificacion: dos letras nombre, dos letras apellido y fecha de nacimiento
